# Discrepancies in Race and Ethnicity Documentation: a Potential Barrier in Identifying Racial and Ethnic Disparities

**DOI:** 10.1007/s40615-016-0283-3

**Published:** 2016-09-08

**Authors:** M. Magaña López, M. Bevans, L. Wehrlen, L. Yang, G. R. Wallen

**Affiliations:** National Institutes of Health (NIH), Clinical Center, 10 Center Drive, Room 2B01, Bethesda, MD 20892 USA

**Keywords:** Race, Ethnicity, Demographic collection, Health disparities

## Abstract

**Background:**

Data collection on race and ethnicity is critical in the assessment of racial disparities related to health. Studies comparing clinical and administrative data show discrepancies in race documentation and attribution.

**Methods:**

Self-reported data from two studies were compared to demographics in the electronic health record (EHR) extracted from the Biomedical Translational Research Information System (BTRIS) repository. McNemar and Bhapkar analyses were conducted to quantify the agreement of ethnicity and race between self-reported and EHR data. Pearson’s chi-square tests were used to explore the relationship between acculturation, length of time in the USA, country of residence, and how individuals self-reported their race.

**Results:**

The sample (*n* = 280) was predominantly female (52.1 %), with a mean age of 47 (SD ± 13.74), mean years in the USA were 12.8 (SD ± 11.67) and the majority were born outside of the USA. (55.6 %). Those who self-identified as Hispanic (*n* = 208) scored a mean of 5.5 (SD ± 3.07) on the short acculturation scale (SAS) that ranges 4 to 20; lower scores indicate less acculturation. A significant difference was found between the way race is reported in the electronic medical record and self-reported data among those people who identified as Hispanic, with significant differences in the *white* (*p* < 0.0001) and *other* (*p* < 0.0001) categories.

**Conclusions:**

The misclassification of race is most frequent in those individuals who self-identified as Hispanic. As the Hispanic population in the USA continues to grow, understanding the factors that affect the way that individuals from this heterogeneous population self-report race may provide important guidance in tailoring care to address health disparities.

## Introduction

Ethnicity and race are complex social constructs that influence personal identity and group social relations. Racial and ethnic identification are fluid and specific to place, time, and context [1, 2]. Despite their fluidity, federal ethnic and racial categories serve as the basis to ensure inclusion of minorities in research as well as identify and address health disparities in the US health care system [2]. Inaccurate or inconsistent data stratified by race and ethnicity can impede analysis needed to identify improvements in health care and for the identification of population groups that might be the focus of health interventions to decrease health disparities [3]. A step toward achieving more equitable health care is the systematic collection of accurate data on patient/participant race and ethnicity [4]. In 1977, the Office of Management and Budget (OMB) issued race and ethnicity standards that state that both race and ethnicity data need to be collected when developing federal reports [5]. The primary reason for standardizing the categories of ethnicity and race is to enable consistent comparison or aggregation of data across multiple sources [3]. The Office of Management and Budget currently allows two formats for the race and Hispanic ethnicity questions. The first combines both race and Hispanic ethnicity into a single question. The Office of Management and Budget explicitly prefers, however, the second option which asks about race and ethnicity in two separate questions with the Hispanic ethnicity question being asked first [3]. Even with a standardized system, research studies report inconsistencies in the way that race and ethnicity are reported [1, 6, 7]. These results indicate the inadequacy of current reporting systems and illustrate that other collection options should be considered to ensure that race and ethnicity are being captured accurately.

Studies specifically investigating race and ethnicity found differences in self-reported and administrative data [6, 7]. Concordance between self-reported responses and electronic health record ranged between 39 and 97 %, highlighting discrepancies across datasets particularly in those who identified as a racial or ethnic minority [1, 7, 8]. Boehmer and colleagues concluded that race and ethnicity data were more frequently incorrect for patients who did not identify as White [6]. Inconsistencies of race and ethnicity were reported between the birth and death of US infants; there was higher agreement for those who identified as White (1.2 %) and Black (4.3 %) and lower for those identified as Hispanic (30.3 %) [8]. Lee and colleagues analyzed the agreement of race and ethnicity between cancer registries and an electronic medical record and found that there was only complete agreement in 39.2 % of pairs; pairs with “black” data value labels had the highest agreement (95.3 %) and pairs with “other” data value labels had the lowest agreement across sources (11.1 %) [9]. The assessment of the validity of race/ethnicity in Medicare databases found that those who were recorded as White and Black in the Medicare electronic health record had better concordance with their self-report data than those who were identified as Hispanic, Asian, Pacific Islanders, American Indians, and Alaskan Natives (39–60 % agreement) [1]. An analysis comparing the classification of individuals by self, proxy, funeral director, and interviewer concluded that how individuals are identified varies over time and by method of ascertainment, as well as the relationship between the person classifying and person classified [6]. Lastly, the source of demographic data on race and ethnicity influences the assessment of the study’s outcome [6]. The level of disagreement and the differences in assessment suggest that the estimates of racial/ethnic counts are dependent on the source.

This paper presents an analysis that was undertaken to evaluate hypothesized discrepancies in the self-reported and medical record documentation of racial and ethnic minority participants in two National Institutes of Health (NIH) Clinical Center intramural clinical trials. Investigators reported that those who identified as Hispanic in the ethnicity question were not answering or were unsure on what they should put in the race category. We further hypothesized that there were higher discrepancies in the reporting of race and ethnicity particularly for those who identified as Hispanic compared to those who identified as non-Hispanic.

## Methods

Data were obtained from two studies previously conducted within the NIH Clinical Center Intramural Research Program; the *Health Beliefs and Health Behavior Practices Among Minorities with Rheumatic Diseases* (HBS) (NCT#0069342) and the *Prospective Assessment of Functional Status, Psychosocial Adjustment, Health Related Quality of Life and the Symptom Experience in Patients Treated with Allogeneic Hematopoietic Stem Cell Transplantation* (FAQS) (NCT#00128960). Baseline self-reported race, ethnicity, residency, length of time in the USA, and level of acculturation data were obtained from the HBS (*n* = 109), an exploratory study designed to examine health beliefs and behaviors of patients receiving care at the National Institute of Arthritis and Musculoskeletal and Skin Diseases (NIAMS) Community Health Center [10, 11] and the FAQS study (*n* = 171), a longitudinal study of a diverse group of allogeneic stem cell transplant survivors [12, 13]. The self-reported data from both studies was combined into one database and then compared to demographic information obtained by hospital admission representatives when creating individual electronic health records (EHR). Admission demographic data was exported from the EHR using the NIH Biomedical Translational Research Information System (BTRIS), a clinical research data repository available to NIH Clinical Center researchers [14].

In order to account for the differences in the way that researchers and admission clerks categorized the race options that did not fit in the standard categories of White, Black/African American, Asian/Pacific Islander, Native American, and Multiple Races, we combined some options. All of those who reported “Other,” “Don’t Know,” and “Unknown Race” were combined to make a category of “Other.” We only compared the categories of those cases that had no missing responses in both databases. Table 1 lists the questions related to race and ethnicity including the categories used by each study and in the EHR.

**Table 1:** Questionnaires used to collect demographic information

Variables	HBS	FAQS	EHR
Race	Which one or more of the following would you say is your race? • White • Black or African America • Asian • American Indian • Other • Don’t Know	Which one or more of the following would you say is your race? • White • Black or African American • American Indian & Alaska Native • Native Hawaiian & other Pacific Islander • Asian • Other	• White • Black/African American • Asian/Pacific Islander Native American • Multiple Races • Unknown
Ethnicity	Are you Hispanic or Latino? • Yes • No • Don’t Know	Hispanic or Latino • Yes • No	Hispanic/Latino • Yes • No
Country of origin	Open-ended question: country of origin_______________	In what region were you born? • USA • Canada • Mexico • Central America • South America • Asia • Europe • Africa • Other	NA
Time in USA	Length of time in the USA	How long (years)	NA
Country of current residence	NA	What region do you reside in currently? • United States • Canada • Mexico • Central America • South America • Asia • Europe • Africa • Other	NA
Acculturation	Please tell us how much Spanish and English you use on a daily basis. Selecting the number 1 would mean that you speak only Spanish and the number 5 would mean only English. Choosing the number 2 would mean that you speak Spanish 75 % of the time and English 25 % of the time. On a scale of 1 to 5. A) In which language do you feel most comfortable speaking? B) With friends what language do you prefer to speak? C) In what language do you think? D) At home, what language do you speak?	Same as HBS	NA

*NA* not applicable

### Statistical Analysis

Frequency and percentages were reported for categorical variables; mean, standard deviation of median, and range were reported for continuous variables. Bhapkar tests were used to assess whether there were overall significant differences between self-reported and EHR ethnicity and race classifications for each participant. McNemar tests were used to determine whether there were significant differences between self-reported and EHR ethnicity and race attributions for each category. Bonferroni adjustments were used to account for multiple testing. Pearson’s chi-square tests or Kruskal-Wallis tests were used to test whether acculturation, length of time in the USA, and country of residence affected the way Hispanic participants reported their races. Data analyses were performed using SPSS and MH Program [15, 16]. A *p* < 0.05 was considered significant.

## Results

Participants were predominantly female (52.1 %) and the mean age was 47 (±13.74) years old (see Table 2). About 59 % were identified as non-Hispanic through self-report and the EHR. Almost half of the participants (48.2 %) self-reported as White, while only one third (33.2 %) were classified as White in the EHR. The majority (55.6 %) were born outside of the USA, and some (21 %) still lived outside of the USA at the time of their interviews. Those that lived in the USA had been there for approximately 12 years (±10.67). On a scale from 4 to 20, their mean acculturation score using the short acculturation scale (SAS) of those who identified as Hispanic was 5.5 (±3.07); lower scores indicate less acculturation [17]. Acculturation (the process of learning and adapting to a new culture) was measured by obtaining the proxy measures: length of time in the USA and English-language proficiency [18]. About a third (37.9 %) were interviewed for the studies in Spanish.

**Table 2:** Demographic information from HBS and FAQS study and combined

	HBS	FAQS	Combined
Gender			
Male	27 (24.8 %)	107 (62.6 %)	134 (47.9 %)
Female	82 (75.2 %)	64 (37.4 %)	146 (52.1 %)
Self-reported ethnicity			
Hispanic/Latino	46 (42.2 %)	69 (40.4 %)	115 (41.1 %)
Non-Hispanic/Latino	63 (57.8 %)	102 (59.6 %)	165 (58.9 %)
Self-reported race			
American Indian	2 (1.8 %)	0 (0 %)	2 (0.7 %)
Asian	3 (2.8 %)	18 (10.5 %)	21 (7.5 %)
Black/African American	40 (36.7 %)	15 (8.8 %)	55 (19.6 %)
White	46 (42.2 %)	89 (52.0 %)	135 (48.2 %)
Other	15 (13.7 %)	48 (28.1 %)	63 (22.5 %)
Interview language			
English	68 (62.4 %)	106 (62 %)	174 (62.1 %)
Spanish	41 (37.6 %)	65 (38 %)	106 (37.9 %)
Place of birth			
United States	52 (47.7 %)	71 (41.5 %)	123 (44.4 %)
Latin America	48 (44 %)	60 (34.1 %)	105 (37.9 %)
Other countries	9 (8.3 %)	40 (23.4 %)	49 (17.7 %)
Age	51.27 ± 13.22	44.53 ± 13.45	47.15 ± 13.74
Acculturation score^a^	5.8 ± 3.90	5.28 ± 2.38	5.5 ± 3.07
Years in the USA	13.16 ± 11.75	12.16 ± 9.16	12.77 ± 10.67
Current country of residence			
United States	109 (100 %)	109 (65.3 %)	218 (78.9 %)
Latin America	0 (0 %)	40 (23.9 %)	40 (14.5 %)
Other countries	0 (0 %)	18 (10.8 %)	18 (6.5 %)

^a^Acculturation score was calculated using a short acculturation scale (SAS) that ranges from 4 to 20; lower scores indicate less acculturation. The only SAS scores that were counted in these were those who self-identified as Hispanic [17]

McNemar and Bhaphkar analysis, noted in Table 3, suggested that there were no significant differences in the reporting of Hispanic/Latino ethnicity between self-reported and electronic health records (*p* = .0588), meaning that most of those who self-identified as Hispanic in the studies (*n* = 280) also were identified as Hispanic in the EHR data, and those who self-identified as non-Hispanic in the research studies also were identified as non-Hispanic in the EHR data.

**Table 3:** Comparison of self-report and administrative assessed race/ethnicity data

		Frequency (*n*)		
Data characteristics	Category	BTRIS	Self-report	*p* value
Ethnicity	Hispanic	109	114	*p* = 0.1797
	Non-Hispanic	164	164	*p* > 0.9999
Race	Other	100	61	*p* < 0.0001
	White	93	135	*p* < 0.0001
Race (Hispanics Only)	Other	103	56	*p* < 0.0001
	White	7	55	*p* < 0.0001
Race (non-Hispanics Only)	Other	2	6	*p* > 0.9999
	White	86	84	*p* > 0.9999

McNemar tests were done to assess *p* value

There were statistically significant differences, however, in the way that race was reported when comparing the same patients’ (*n* = 274) self-reported information to their EHR data as noted in Table 3. Bhapkar tests of overall marginal homogeneity concludes that the two datasets were significantly different overall (*p* < 0.0001). McNemar tests, which examined at the differences in each category, found that there were significant differences in the *white* (*p* < 0.0001) and *other* (*p* < 0.0001) categories using a Bonferroni-adjusted significance criterion.

By running the same tests but looking at those participants who identified as Hispanic/Latino and non-Hispanic/Latino separately, we were able to determine that the statistically significant discrepancies in race reporting are only seen in the population who identifies as Hispanic/Latino. No significant discrepancies in race reporting were found across datasets for non-Hispanics/Latino participants. Table 3 summarizes the differences in (1) race overall, (2) race of Hispanics/Latinos only, and (3) race of non-Hispanics/Latinos only.

To understand why these discrepancies may exist, Pearson’s chi-square tests or Kruskal-Wallis tests were used to test whether acculturation, length of time in USA, and country of residence affect the way Hispanics reported their races. Acculturation scores and length of time in the USA had no significance in the way that self-reported Hispanics/Latinos identify in the race category in both databases. Acculturation scores were analyzed using all of the race categories (*p* = 0.382) and were dichotomized to white/non-white (*p* = 0.635). Time in the USA was also analyzed using all race categories (*p* = 0.473) and were dichotomized to white/non-white (*p* = 0.637). Country of residence, however, is shown to be significant in affecting the way that Hispanic/Latino participants’ (*n* = 110) race was categorized in the EHR and in the self-reported database (*p* < 0.0001). The same statistically significant results existed when dichotomizing country of residence USA/non-USA as well as when examining the USA, Latin American, and Other countries separately.

## Discussion

Collecting demographic information is a federal mandate, but there may be issues with the way data are collected. The findings from these analyses add to the existing body of literature which demonstrates that inconsistencies in ethnicity and race reporting are prevalent. Our findings are further supported by data from the most recent census that concludes that a growing percentage of Americans do not fill out the race category on provided questionnaires [3, 19]. Of those that do provide an answer, many people feel as though they do not belong in the predefined categories. As many as 19 million census respondents selected “some other race” to describe themselves, 97 % of which were Hispanic [19]. A separate study that used birth certificate data concluded that two thirds of the 15,074 mothers of Hispanic ethnicity reported their race as “some other race” [20]. Reasons for this phenomenon may be that Hispanic/Latino communities may be unsure of how to identify themselves in the race question or do not identify with the categories provided.

Our results demonstrate that there are significant differences in the way that race is recorded between self-report and EHRs or administrative databases. As others have described, we found significant discrepancies in race reporting particularly among participants who self-identify as Hispanic/Latino. Race and ethnicity are common, yet often unvalidated, variables used to compare outcomes across different racial and ethnic groups [21]. Research that incorporates race and ethnicity, if accurately captured, has the potential to give clarity to the understanding of factors that affect disease and health since individuals in the same ethnic group may share linguistic, dietary, religious traits, and potentially similar outlooks on health and health care [22]. Results from this analysis solidify how discordant the reporting of race and ethnicity is among the same group of participants when comparing two different clinical trial data sources. These discrepancies call into question the reliability of health disparity analyses that use race and ethnicity as the primary variables of interest. Unreliable data collection on race and ethnicity can lead to a misunderstanding of disease burden and therefore result in the under appropriation of funds to combat health disparities. In order to accurately capture this vital information, the way in which we collect information on race and ethnicity may need to be re-evaluated.

It is important to highlight the fact that the Hispanic/Latino participants had the highest inconsistencies in the reporting of race in both databases with the most common responses “White,” “Other,” “Unknown Race,” and “Don’t Know.” Our study builds on previous research which has shown that barriers may exist in effectively capturing the heterogeneity of Hispanic/Latino subpopulations using the OMB categories [3]. Many Hispanic/Latino individuals prefer to self-identify using their specific ancestry as opposed to the general category Hispanic/Latino on the ethnicity question [23, 24]. Furthermore, a survey conducted by the Pew Center concluded that two thirds of Hispanic adults say being Hispanic is part of their racial background and therefore may become confused about what box to check in the race category [25]. Preliminary findings from one study suggest that this issue can be ameliorated by combining race and ethnicity into a single question which is under consideration for 2020 [19]. However, the study by Krongstad and colleagues found that this change did not result in a reduction of the proportion of population identifying as Hispanic and the selection of “some other race” declined to less than 1 % of respondents [19].

Inconsistencies in race reporting may also be due to lack of training and enforcement when collecting demographic information. Institutional incentives and the enforcement of data collection policies are necessary to drive the uniform day-to-day collection of race and ethnicity information from patients enrolled in clinical trials. Researchers who are interested in understanding the health outcomes in various underrepresented groups should consider how they will accurately and consistently ask for this demographic information. Moreover, it is critical for researchers to understand how the demographic information on race and ethnicity will be used in answering scientific questions of interest. Participants are often asked to report race and ethnicity as a social identity construct while researchers seeking to understand biological predictions in an era of precision medicine may find that their outcomes would be better quantified using ancestry as the predictive variable of choice [21]. Additionally, many health disparities are explained by social determinants of health such as income, acculturation, and education level. Researchers should consider if these variables are better proxies for them to understand the development of a health condition or health outcome within social contexts. Our research demonstrated that the country of residence was significant in determining how participants self-identified in the race category, thus suggesting that the country of origin may serve as an additional variable from which to understand the factors that may affect the way an individual self-selects their race and ethnicity. As the Hispanic population in the USA continues to grow, understanding the factors that affect the way that individuals from this heterogeneous population self-report race may provide important guidance in tailoring care to address health disparities.

Future directions should include a comparative analysis to see if self-reported and EHR data yield similar results when evaluating disparities with specific health outcomes. More research should also be done to understand what variables would be more consistent and accurate in understanding the prevalence of diseases among diverse groups of people rather than using race and ethnicity alone. A growing body of evidence suggests discrepancies exist in the recording of race and ethnicity where attribution depends on the manner in which it is collected, the interpretation from participant, and the options available to individuals for self-categorization.

## Limitations

This analysis was done using information from participants who consented to participate in two separate research studies conducted at the National Institutes of Health Clinical Center, and results may not be generalizable to the general population. While both studies collected similar demographic questions and categories for self-categorization, there were slight differences in response options. For example, admission clerks informed us that they first ask a patient if they are Hispanic/Latino (yes/no). Then, they ask what race they are and give them six options: White, Black/African American, Asian/Pacific Islander, Native American, Multiple Races, or Unknown. However, the data output from the electronic health record also included “Don’t Know.” Therefore, based on their options, we assumed that “Don’t Know” and “Unknown” would be the same and thus combined the categories. However, we analyzed the data as combined categories and as separate categories, and both revealed the same significant results. Previous reports indicate that minority participation in clinical trials is approximately 30 % with Hispanic participants ranging from 1 to 7.6 % in all trials and 2.5 % in cancer trials [26, 27]. Similarly, most recent reports from the NIH found that in 2014, minorities accounted for 30 % of participants in all NIH funded trials; of those, 8.3 % were Hispanic [28]. However, there were *n* = 69 (40.4 %) Hispanic participants in the FAQS study [12, 13] and *n* = 46 (42.2 %) in the HBS study [11], well above ‘typical’ Hispanic participation for NIH clinical trials, which may limit generalizability of these findings in populations with fewer Hispanic participants.
